# Vascular regulation of adult neurogenesis under physiological and pathological conditions

**DOI:** 10.3389/fnins.2014.00053

**Published:** 2014-03-17

**Authors:** Masato Sawada, Mami Matsumoto, Kazunobu Sawamoto

**Affiliations:** Department of Developmental and Regenerative Biology, Nagoya City University Graduate School of Medical SciencesNagoya, Japan

**Keywords:** adult neurogenesis, ventricular-subventricular zone, neural stem cell, neuronal migration, blood vessel, brain injury, ischemia, brain regeneration

## Abstract

Neural stem cells in the mammalian adult brain continuously produce new neurons throughout life. Accumulating evidence in rodents suggests that various aspects of adult neurogenesis, including the genesis, migration, and maturation of new neurons, are regulated by factors derived from blood vessels and their microenvironment. Brain injury enhances both neurogenesis and angiogenesis, thereby promoting the cooperative regeneration of neurons and blood vessels. In this paper, we briefly review the mechanisms for the vascular regulation of adult neurogenesis in the ventricular-subventricular zone under physiological and pathological conditions, and discuss their clinical potential for brain regeneration strategies.

## Introduction

**Figure 1:**
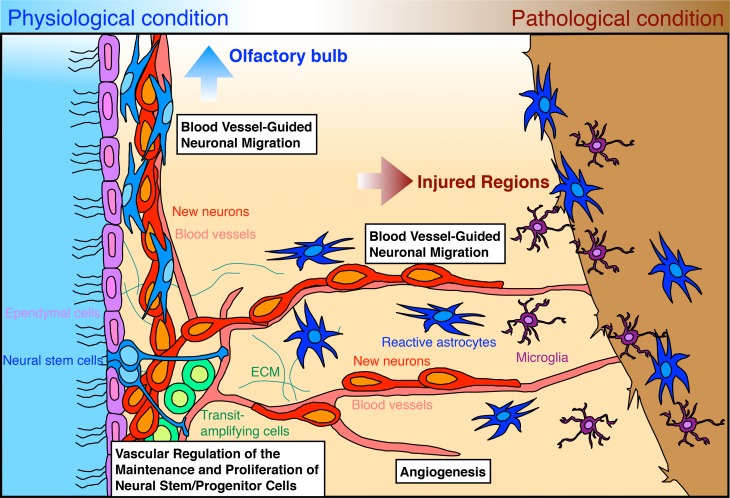
**Vascular regulation of adult neurogenesis in the V-SVZ under physiological and pathological conditions**. Neural stem cells (blue) and transit-amplifying cells (green) contact blood vessels (pink) in different manners and generate new neurons (red). Blood vessel-derived factors regulate the maintenance and proliferation of neural stem cells and transit-amplifying cells within the V-SVZ. The generated new neurons form chains and migrate along blood vessels toward the OB. Ependymal cells lining the wall of the lateral ventricle are shown in light purple. In the injured brain, neurogenesis and angiogenesis are dynamically upregulated. The injury results in increased levels of growth and trophic factors, which control both angiogenesis and various aspects of neurogenesis (the genesis, migration, or maturation of new neurons). Some new neurons generated in the V-SVZ migrate in chains along blood vessels toward the injured regions. New neurons migrating toward injured regions are attracted by various chemokines secreted from reactive astrocytes (dark blue) and microglia (dark purple).

Embryonic development of the nervous and vascular systems in mammals proceeds in a coordinated manner, in which neurogenesis and angiogenesis are regulated by common instructive cues and tightly dependent on each other, leading to the formation of parallel networks (Segura et al., 2009; James and Mukouyama, 2011). In adults, while the vascular network is completely developed, neurogenesis still occurs in two restricted brain regions: the ventricular-subventricular zone (V-SVZ) in the lateral wall of the lateral ventricles and the subgranular zone of the dentate gyrus in the hippocampus; at these sites, neural stem cells continuously generate new functional neurons (Lledo et al., 2006; Zhao et al., 2008; Ihrie and Alvarez-Buylla, 2011; Fuentealba et al., 2012). Palmer et al. (2000) reported that neural stem cells and their progenies are closely associated with blood vessels, raising the possibility that adult neurogenesis is controlled by factors derived from blood vessels and their microenvironment (Goldberg and Hirschi, 2009; Goldman and Chen, 2011). Following brain tissue injury, both neurogenesis and angiogenesis are upregulated, and these processes are tightly coupled in the endogenous mechanism for brain regeneration (Greenberg and Jin, 2005; Kaneko and Sawamoto, 2009; Massouh and Saghatelyan, 2010). The coordinated neurogenesis and angiogenesis processes observed following brain injury appear to recapitulate the events occurring in the embryonic and early postnatal stage. Thus, blood vessels are likely to be important in brain regeneration as they are in its development. In this paper, we briefly review the latest findings on the mechanisms of vascular regulation of adult neurogenesis in the V-SVZ under physiological and pathological conditions (Figure 1), and discuss their clinical potential for brain regeneration strategies.

## Relationship between neurogenesis in the V-SVZ and blood vessels under physiological conditions

In the V-SVZ, neural stem cells and transit-amplifying cells, which arise from the stem cells and eventually differentiate into neural cells, interact with blood vessels differently (Mirzadeh et al., 2008; Shen et al., 2008; Tavazoie et al., 2008). In contrast to most blood vessels in the brain, capillaries in the V-SVZ are not completely enwrapped by pericytes and astrocytic endfeet, and thus form a unique, incomplete blood-brain barrier (BBB), which is permeable to blood-derived small molecules (Tavazoie et al., 2008; Lacar et al., 2012b). Neural stem cells extend a long basal process that makes contact with these capillaries (Mirzadeh et al., 2008; Lacar et al., 2011), while the transit-amplifying cells contact vascular endothelial cells directly with their cell body at the incomplete BBB (Tavazoie et al., 2008). Several studies have suggested that vascular cells including pericytes have the potential to generate neural cells (Palmer et al., 2000; Yamashima et al., 2004; Yokoo et al., 2005; Dore-Duffy et al., 2006; Paul et al., 2012), and evidence suggests that the branch points of blood vessels might provide a preferential environment for neurogenesis in the V-SVZ (Shen et al., 2008). Thus, the vascular microenvironment in the V-SVZ might promote the ability of neural stem cells and transit-amplifying cells to proliferate and generate new neurons.

In olfaction, new neurons generated in the V-SVZ destined for the olfactory bulb (OB) also associate with blood vessels in the rostral migratory stream (RMS). At the neonatal stage, new neurons migrate along blood vessels not only to the OB (Bozoyan et al., 2012) but also to the cerebral cortex (Inta et al., 2008; Le Magueresse et al., 2012). Although blood vessel-guided neuronal migration toward the cerebral cortex gradually decreases during postnatal development, probably due to the decrease in blood vessel density in the corpus callosum (Le Magueresse et al., 2012), new neurons continue to migrate along blood vessels in the RMS and OB even in adulthood (Bovetti et al., 2007; Snapyan et al., 2009; Whitman et al., 2009). During blood vessel-guided migration, the new neurons directly contact astrocytic processes and vascular endothelial cells (Bovetti et al., 2007; Whitman et al., 2009; Le Magueresse et al., 2012). These studies suggest that new neurons generated in the V-SVZ utilize blood vessels as a migratory scaffold in the adult brain.

## Relationship between neurogenesis in the V-SVZ and blood vessels under pathological conditions

Brain injuries such as ischemic stroke and traumatic injury cause cell death, disruption of blood vessels, and inflammation, leading to functionally irreversible damage. Recent studies in rodents show that after brain injury, new neurons generated in the V-SVZ migrate toward the injured region and differentiate into mature neurons, suggesting that the endogenous neural stem cells in the adult V-SVZ have clinical potential for brain regeneration (Arvidsson et al., 2002; Parent et al., 2002; Yamashita et al., 2006).

The first responses after brain injury include increased angiogenesis around the injured region and neural progenitor cell proliferation in the V-SVZ. In an ischemic stroke model such as middle cerebral artery occlusion (MCAO), responsive angiogenesis remodels the disrupted blood vessel network in the injured striatum, several days to two weeks after the injury (Thored et al., 2007). In addition, increased proliferation of the neural stem cells and transit-amplifying cells in the V-SVZ occurs. Unlike the case under physiological conditions, the proliferation of these cells contributes not only to neurogenesis and oligodendrogenesis, but also to reactive astrogliosis (Li et al., 2010; Zhang et al., 2011; Benner et al., 2013).

The second response after injury is the appearance of new neurons at the injured region (Arvidsson et al., 2002; Parent et al., 2002). These new neurons are generated from neural stem cells in the V-SVZ and migrate toward the ischemic striatum after MCAO (Yamashita et al., 2006). The neuronal migration toward the injured region shares two important features with that in the RMS: first, the migrating new neurons form chain-like cell aggregates (Arvidsson et al., 2002; Parent et al., 2002; Yamashita et al., 2006) and second, they migrate along blood vessels (Zhang et al., 2009; Kojima et al., 2010; Saha et al., 2013). During the blood vessel-guided migration, new neurons are observed to be frequently associated with thin astrocytic processes aligned close to the blood vessels (Yamashita et al., 2006). Inhibiting angiogenesis decreases the number of new neurons in injured regions (Taguchi et al., 2004; Ohab et al., 2006; Cayre et al., 2013), suggesting that newly generated blood vessels play a role in neuronal regeneration. However, since new neurons migrate along both pre-existing and newly generated blood vessels after MCAO (Kojima et al., 2010), both old and new blood vessels appear to act as the migratory scaffold for new neurons moving toward injured regions. Notably, in the ischemic striatum, the new neurons migrating along blood vessels frequently make a “U-turn” when they reach the branch point of a blood vessel and go back toward the V-SVZ, and jump from one blood vessel to another; such turns are not observed for the new neurons in the RMS (Kojima et al., 2010; Grade et al., 2013). Thus, remodeling the blood vessel network in injured regions and regulating the direction of new neuron migration could improve the efficiency of blood vessel-guided neuronal migration and neuronal regeneration.

## Mechanisms for vascular regulation of adult neurogenesis under physiological and pathological conditions

### Blood flow and cerebrospinal fluid flow

Local changes in blood flow in the V-SVZ, in which neural stem cells and transit-amplifying cells are involved (Lacar et al., 2012a,b), alter the levels of metabolic and gas molecules around the blood vessels, thereby affecting neurogenesis in the V-SVZ. For example, ATP regulates the proliferation of neural stem cells and transit-amplifying cells (Lin et al., 2007; Suyama et al., 2012). Nitric oxide (NO) and oxygen (O_2_) are also thought to affect the proliferation of neural stem cells (Matarredona et al., 2005; Panchision, 2009). In addition to these small molecules, fluid dynamics could also regulate the active transport of blood-derived factors such as insulin-like growth factor-1 (IGF-1) (Nishijima et al., 2010), to control neurogenesis in the V-SVZ. These studies collectively suggest that cell proliferation in the V-SVZ is modulated by changes in blood flow.

Interestingly, a recent study showed that subarachnoid cerebrospinal fluid (CSF) enters the brain parenchyma along the spaces surrounding arteries and is excreted along the spaces surrounding veins (Iliff et al., 2012). Ventricular CSF contains several morphogens and growth factors that are known to control neuronal migration, such as Slit (Sawamoto et al., 2006) and Sonic hedgehog (Shh) (Angot et al., 2008). In addition, Shh is increased in the CSF after injury and plays a role in neural regeneration (Sirko et al., 2013). If the blood vessels and their surrounding spaces provide not only a scaffold but also CSF-derived guidance cues, they might promote the efficient migration of new neurons in the adult brain.

These studies indicate that the fluid dynamics of blood and CSF might be related to neurogenesis in the V-SVZ under physiological conditions. In addition, blood flow-mediated mechanical forces might affect the state of neural stem cells and their progenies, as reported in the hematopoietic system (Adamo et al., 2009). Although these flows are likely to be disrupted by various brain injuries, their effects on neurogenesis in the V-SVZ of the injured brain have not been elucidated. It is tempting to speculate that the fluid dynamics of blood and CSF help control neurogenesis in response to changes in body circulation and brain activity.

### Growth/trophic factors

The maintenance and proliferation of neural stem cells and their progenies are controlled by soluble factors released from blood vessels (Louissaint et al., 2002; Shen et al., 2004). For example, pigment epithelium-derived factor (PEDF), which is secreted from ependymal cells and vascular endothelial cells in the V-SVZ, promotes the self-renewing cell division and multipotency maintenance of neural stem cells by enhancing Notch-dependent transcription (Ramirez-Castillejo et al., 2006; Andreu-Agullo et al., 2009). Vascular endothelial cell-derived betacellulin, which belongs to the epidermal growth factor (EGF) family, regulates the proliferation of both EGFR-expressing neural stem/transit-amplifying cells and ErbB4-expressing new neurons (Gomez-Gaviro et al., 2012). Vascular endothelial growth factor (VEGF) and angiopoietin-1 (Ang-1), which are potent angiogenic growth factors, can also stimulate cell proliferation in the V-SVZ (Jin et al., 2002; Sun et al., 2006; Rosa et al., 2010). On the other hand, in older adult to aged mice, transforming growth factor-β (TGF-β) increases in vascular endothelial cells, and mediates the apoptosis of neural stem cells in the aged V-SVZ (Pineda et al., 2013). Taken together, these studies indicate that under physiological conditions, blood vessel-derived growth factors and angiogenic factors control the maintenance and proliferation of neural stem/progenitor cells in the V-SVZ.

Evidence suggests that brain-derived neurotrophic factor (BDNF) derived from vascular endothelial cells promotes blood vessel-guided neuronal migration in both the RMS and the ischemic striatum. In the RMS, astrocytes play an important modulatory role in the blood vessel-guided neuronal migration by controlling the BDNF level (Snapyan et al., 2009). Similarly, in the ischemic striatum, endothelial-derived BDNF is captured by reactive astrocytes (Grade et al., 2013). Thus, the endothelial BDNF-astrocyte-neuron interaction may be a common mechanism for blood vessel-guided neuronal migration in the adult brain under physiological and pathological conditions.

Ischemic stroke results in the increased expression of several factors, including VEGF, Ang-1, and Netrin-1, that exert both neurogenic and angiogenic activities (Hayashi et al., 2003; Sun et al., 2003; Ohab et al., 2006; Liu et al., 2007a; Thau-Zuchman et al., 2010; Cayre et al., 2013). Inhibiting VEGF signaling by a VEGFR-2 inhibitor or an anti-VEGF blocking antibody reduces the injury-induced angiogenesis in a rodent model of stroke (Shimotake et al., 2010). On the other hand, exogenous VEGF injection or VEGF overexpression promotes injury-induced angiogenesis, neural progenitor proliferation in the V-SVZ, and new neuron migration to and survival at the injured regions, which contribute to functional recovery (Sun et al., 2003; Wang et al., 2007; Thau-Zuchman et al., 2010). Since VEGF can promote the proliferation, migration, and differentiation of V-SVZ cells *in vitro* (Zhang et al., 2003; Barkho et al., 2008), it is possible that VEGF directly influences each of these processes following injury. On the other hand, it is also possible that VEGF-induced angiogenesis results in increased levels of blood vessel-derived factors that promote neurogenesis, as it is observed in the adult songbird brain, where testosterone-mediated increase in VEGF levels induces the expression of BDNF by endothelial cells that in turns promotes neurogenesis (Louissaint et al., 2002).

Collectively, these studies show that blood vessel-derived factors control various aspects of neurogenesis in the V-SVZ under physiological and pathological conditions. It is possible that some of these factors affect both neurogenesis and angiogenesis directly or indirectly. An increased understanding of the mechanisms by which angiogenesis and neurogenesis are regulated by these factors may lead to new strategies for brain regeneration.

### Chemokines

Stromal cell-derived factor-1 (SDF-1) has different roles in neurogenesis under physiological vs. pathological conditions. Under physiological conditions, a high level of SDF-1 derived from ependymal cells maintains neural stem cell quiescence, whereas SDF-1 from vascular endothelial cells enhances the activated state of both activated neural stem cells and transit-amplifying cells, thereby generating quiescent and activated niches for neural stem cells in the V-SVZ (Kokovay et al., 2010). On the other hand, after ischemic stroke, SDF-1 is secreted from vascular endothelial cells and reactive astrocytes in the injured regions (Ohab et al., 2006; Thored et al., 2006). Blocking C-X-C motif receptor-4 (CXCR-4), which is a receptor for SDF-1, suppresses the migration of new neurons *in vitro* and *in vivo*, suggesting that SDF-1/CXCR-4 signaling promotes neuronal migration in the injured brain (Robin et al., 2006; Thored et al., 2006; Kojima et al., 2010).

Other chemokines, including C-C motif ligand-2 (CCL2), monocyte chemoattractant protein-1 (MCP-1), macrophage inflammatory protein-α (MIP-α), and C-X-C motif ligand-1 (CXCL-1), also increase after injury (Liu et al., 2007b; Yan et al., 2007; Gordon et al., 2009). CCL2 increases in the V-SVZ after MCAO and induces neural progenitor cell differentiation into the neuronal lineage and new neuron migration *in vitro* (Liu et al., 2007b). MCP-1 is secreted from reactive astrocytes and microglia after MCAO, while its receptor C-C motif receptor-2 (CCR-2) is expressed by migrating new neurons in the ischemic striatum (Yan et al., 2007). MCP-1 has attractant activity for migrating new neurons, and the injury-induced migration of new neurons in the ischemic striatum is not observed in MCP-1 or CCR-2 knockout mice (Yan et al., 2007), suggesting that MCP-1/CCR-2 signaling is required for new neuron migration in the injured brain.

Despite their attraction by chemokines, new neurons in the injured brain do not migrate for long distances from the V-SVZ, possibly due to insufficient and irregular chemokine gradients in the injured regions. Thus, supplying strong concentration gradients of chemokines in injured brain regions might attract new neurons, promoting their migration to the injured regions along blood vessels without making U-turns.

### Extracellular matrix

The extracellular matrix (ECM) surrounding blood vessels in the V-SVZ is thought to be produced by vascular endothelial cells, blood vessel-ensheathing pericytes and astrocytes, and neural stem cells and their progenies. Under physiological conditions, an ECM-enriched microenvironment might provide the proper neurogenic milieu for neural stem cells and their progenies in the V-SVZ (Kazanis et al., 2010). A fiber-like basal lamina called fractone is observed in the V-SVZ (Mercier et al., 2002). Heparan sulfate proteoglycan (HSPG), a component of the vascular basal membrane, can anchor bone morphogenetic protein-7 (BMP-7) and promote its inhibitory activity on cell proliferation in the V-SVZ (Lim et al., 2000; Douet et al., 2012). HSPG can also interact with other BMPs, Shh, Wnts, Slits, and several growth factors, and modulate their bioactivities, which regulate neurogenesis in the V-SVZ (Sawada and Sawamoto, 2013). Laminin, another vascular basal membrane component, modulates the interaction between blood vessels and transit-amplifying cells, which express α6β 1 integrin (Shen et al., 2008). SDF-1 enhances the laminin binding of activated neural stem cells and transit-amplifying cells, to maintain the association between these cells and blood vessels (Kokovay et al., 2010).

Although several ECM proteins and their receptors, including tenascin C, αV integrin, and β 3 integrin, increase in the V-SVZ after ischemic stroke (Liu et al., 2007a), their roles in neurogenesis are unclear. Because there are many kinds of ECM components around blood vessels, it is necessary to integrally classify their expression levels and bioactivities under physiological and pathological conditions. It is also important to determine what receptors for ECM components are expressed on neural cells to elucidate the ECM's roles in the relationship between neural stem or progenitor cells and blood vessels.

## Perspective

Adult neurogenesis consists of multiple stages including the genesis, migration, and maturation of new neurons. Recent studies in rodents have demonstrated that blood vessels regulate various aspects of neurogenesis in the V-SVZ under physiological conditions. Although brain injury dramatically changes the environment surrounding the V-SVZ, physiological and regenerative neurogenesis mechanisms depend on common vascular regulations, suggesting that blood vessels have fundamental roles in adult neurogenesis. One limitation of the endogenous neuronal regeneration is the insufficient supply of new neurons migrating into injured regions. Thus, reorganization of the migration scaffold by promoting angiogenesis or transplanting artificial blood vessel-like fibers could be a promising strategy for improving the supply of new neurons to injured regions. Moreover, since the blood flow dynamics in the brain can change, blood vessels may be involved in modifying adult neurogenesis according to the body circulation and brain activity states. Further understanding of the vascular regulations of adult neurogenesis should contribute to the development of new clinical strategies for neuronal regeneration using endogenous neural stem cells in the adult brain.

### Conflict of interest statement

The authors declare that the research was conducted in the absence of any commercial or financial relationships that could be construed as a potential conflict of interest.
